# Commercial Influence on Political Declarations: The Crucial Distinction Between Consultation and Negotiation and the Need for Transparency in Lobbying: Comment on "Competing Frames in Global Health Governance: An Analysis of Stakeholder Influence on the Political Declaration on Non-communicable Diseases"

**DOI:** 10.34172/ijhpm.2021.132

**Published:** 2021-09-11

**Authors:** Astrid Berner-Rodoreda, Albrecht Jahn

**Affiliations:** Heidelberg Institute of Global Health, University of Heidelberg, Heidelberg, Germany.

**Keywords:** Transparency, Lobbying, Private Sector, Political Declaration, NCD, Legislative Footprint

## Abstract

Suzuki and colleagues’ rare and elaborate analysis of the political processes behind the 2018 United Nations (UN) non-communicable diseases (NCD) Declaration discloses various pathways towards influencing global public health policies. Their study should be a wake-up call for further scientific political scrutiny and analysis, including clearly distinguishing between consultations such as UN multi-stakeholder hearings preceding high-level meetings and the actual negotiating and decision making process. While stakeholder positions at interactive hearings are documented and published and thus made transparent, the negotiating process among member states is not publicly known. The extent to which intergovernmental negotiations are influenced at country or regional levels by commercial interests through direct and indirect lobbying outside of public consultations should be given more attention. Lobby registers should be implemented more stringently and legislative footprints required and applied not only to legally binding but also to internationally important documents such as political declarations.

 Comparing the initial draft of the non-communicable disease (NCD) Declaration of 2018 with the final and approved document, Suzuki and colleagues trace the influence of inputs from stakeholder and government groups to the final political declaration yet rightly acknowledge that the final document is negotiated between United Nations (UN) member states not between all stakeholders (p. 3).^1^ They also question if the private industry should be included at all in consultations such as the interactive stakeholder hearing (p. 10).^1^ While we concur with the authors and others that it is problematic to include industries with a potential conflict of interest such as the food and beverage industries in the policy-making of NCDs,^2^ we think a clear distinction should be made between a public stakeholder consultation and the non-public negotiation part of the of the decision making process in terms of inclusion and non-inclusion of stakeholders with a vested interest in the subject matter such as the food and beverage industry in NCDs.

 Consultations serve an important function in hearing concerns by various groups – most importantly those affected but also those with a vested interest in the matter to establish an evidence-base for policies. UN multi-stakeholder hearings and consultations are public and transparent with oral and written statements by stakeholders being made accessible and thus constitute a democratic process that in our view should remain open to all stakeholder groups. The various and diametrically opposed concerns by stakeholders are important to note and to consider for member states in negotiating the final text of a political declaration.

 While the private sector provided only few public comments (9) compared to NGOs and academic institutions (99) (p. 3),^1^ it seemed more successful in influencing the final outcomes of the NCD political declaration as reflected by the non-inclusion of the taxation of sugar-sweetened beverages ^3^ against substantial evidence having been published prior to the NCD High Level Meeting (HLM) that “law can be a cost-effective and affordable means of curbing underlying drivers of the NCD pandemic” (p10).^4^ This poses the question of how the private sector wielded its influence.

## Negotiations by Member States – and (Invisible) Third Parties?

 Member states are free to include civil society actors as well as private industry representatives in their country delegations (https://www.un.org/en/ga/about/ropga/delegt.shtml). These representatives will have privileged access to the country’s government officials and negotiators and can possibly influence government positions if negotiations are still ongoing at the HLM; they can also inform their own stakeholder group. While one can assume that non-profit NGO representatives have the common and global good of health and human rights in mind when trying to influence their own government’s position for these inter-governmental negotiations, this cannot be automatically assumed for companies that exist to make profit and which pursue their own agenda. It may therefore be wise to reconsider eligibility criteria for national delegates to either exclude for profit entities or to restrict these to small and medium sized enterprises. The inclusion of big businesses as partners at UN-level also needs further scrutiny. Rather than seeing it as governments’ task to create ‘enabling environments’ for industry actors, Pingeot calls for the adoption of “more stringent criteria and rules for those who will enter these partnerships and how these actors will be held accountable” (p. 29).^5^

 Political declarations are, however, often finalized before the HLM takes place, and are adopted on the first day of the HLM as happened at the UN General Assembly on September 27, 2018 with the NCD Political Declaration^3^ or on June 8, 2016 with the Political Declaration on HIV.^6^ It is therefore the time between the first draft of the document, a multi-stakeholder consultation and the final wording of the declaration that constitutes the crucial period for intergovernmental negotiations. Yet how much influence other actors and in particular big businesses have on government representatives and policy processes during this period remains unclear.

 Legislation on tobacco taxes and marketing across the globe has shown the various methods used by the industry to influence both public opinion and policy such as generating “alternative evidence,” infiltrating government decision processes by industry actors, paying experts to further the industry’s cause, litigating or threatening to litigate against governments.^7^ The food and beverage industries have been shown to employ similar strategies to the tobacco industry with cross-ownership of companies.^2,8^ The private sector has a long history of resistance to binding political agreements which affect their business operations within and beyond NCDs as has been shown in pharmaceutical companies opposing the essential drug list by the World Health Organization (WHO) in the 1970s^9^; a list which led to a steep decline in drug prices and a doubling of access to essential medicines globally before the turn of the century^10^ and which would not have seen the light of day if pharmaceutical companies had had their way.

 How much sway big business has on the final wording of political declarations remains unclear: some of it may be indirect and compelled by the desire of government officials to protect job security in their countries thereby bowing to industry’s demands, some of it may be due to direct lobbying by the industry. A general lobby register would provide more transparency for the public on who is intervening in what area but is only considered useful if it includes the theme and objective of lobbying alongside the designated public official being lobbied and personal and human resources spent on lobbying.^11^ At EU-level, a so-called legislative footprint is presently voluntary for members of the European Parliament when producing reports, see (https://www.europarl.europa.eu/at-your-service/en/transparency/lobby-groups). “The legislative footprint is a document that would detail the time, person and content of a legislator’s contact with a stakeholder. Published as an annex to legislative reports, it would provide insight into who gave input into draft legislation” (p. 3).^12^ To make this a requirement for all political lobbying at country, regional and UN-level would increase the political transparency of knowing who influenced legislation and political declarations or resolutions. While UN political declarations are recommendations to member states and not legally binding, their political importance at country level and globally is not to be underestimated as they are being used by civil society as reference documents for monitoring progress and for holding governments accountable – two important civil society functions mentioned by Patterson and colleagues in a quest to make progress on overcoming obesity.^13^

## Conclusion

 We concur with Suzuki and colleagues that the UN in its quest to forge sustainable partnerships may have gone too far in upholding the role of private industry and in subscribing to a market-oriented outlook. While we do not support the position that private industry actors should be excluded from preliminary consultations in which all positions are documented, we perceive an urgent need for documenting how member state delegations form their positions, which lobbyists they receive during the negotiating process and which positions they take forward and defend in intergovernmental negotiations. The application of the “legislative footprint” to the negotiation of global political consensus decisions is more than overdue and would allow future research to address the lacunae of how much lobbying takes place behind the scene, by whom and whose position is taken forward in the final version of political declarations.

## Ethical issues

 Not applicable.

## Competing interests

 Authors declare that they have no competing interests.

## Authors’ contributions

 ABR drafted the commentary. AJ reviewed and edited it. The final version was agreed upon and approved by both authors.
